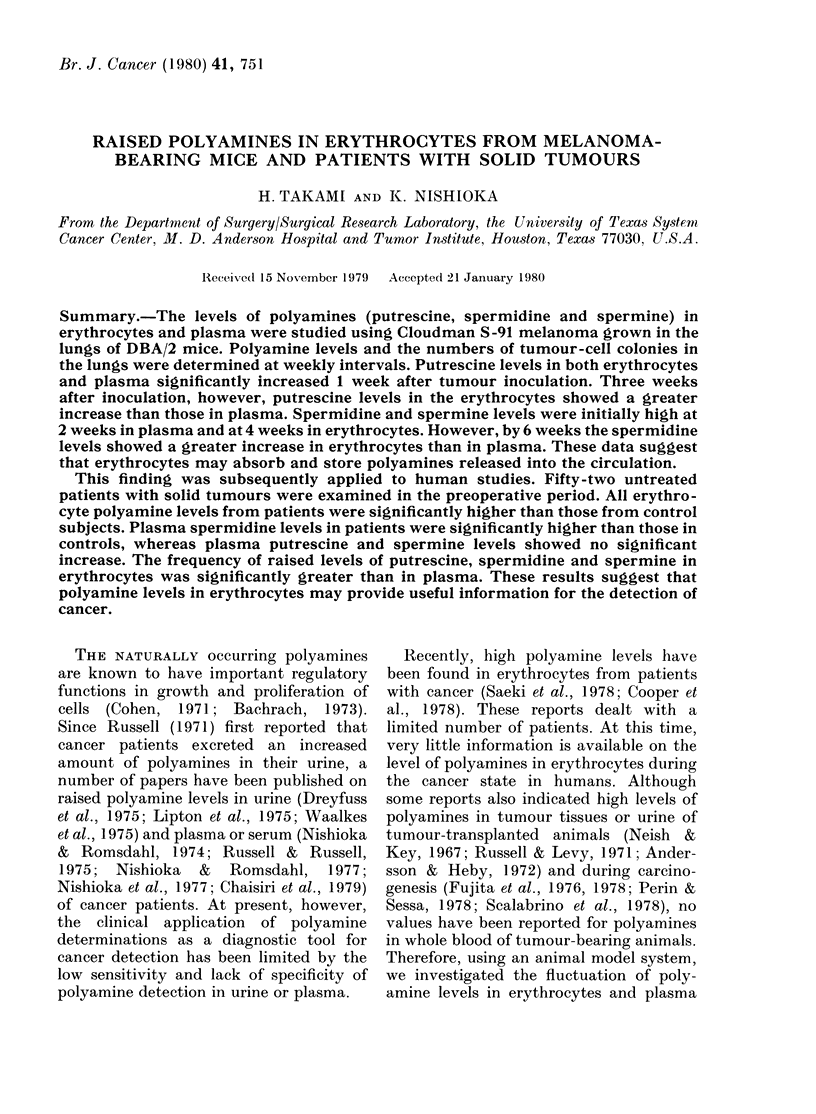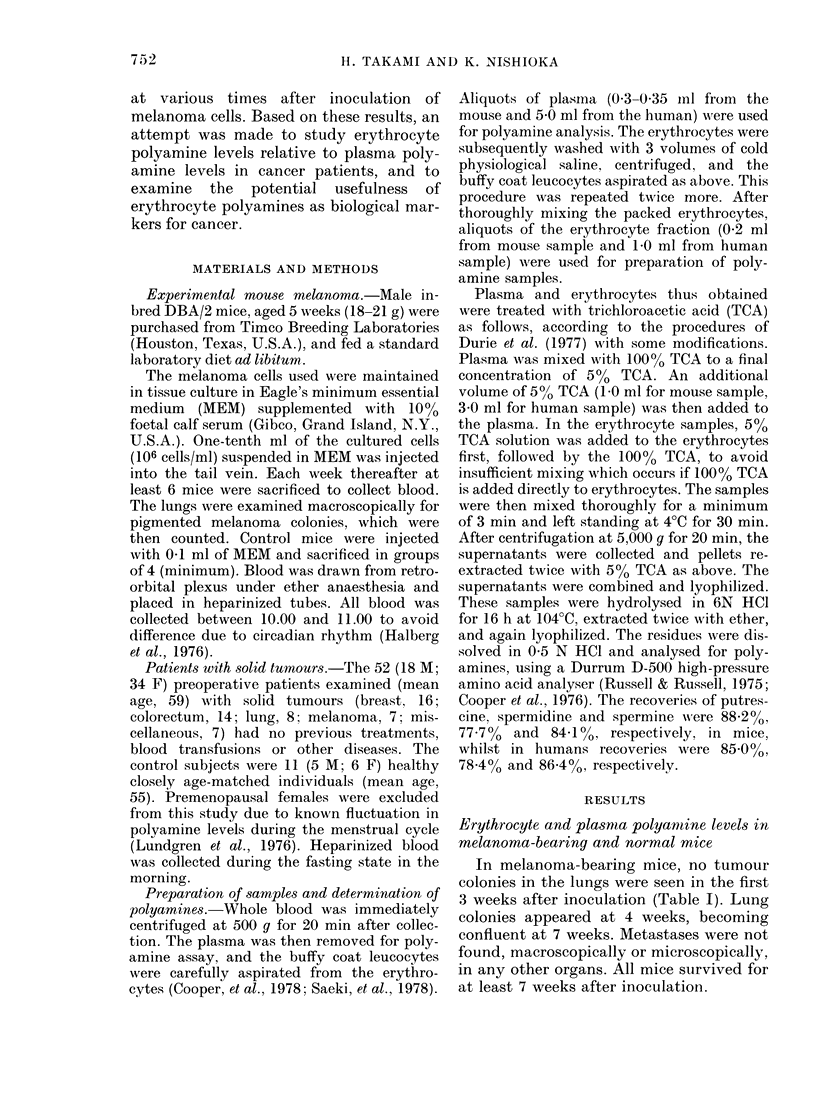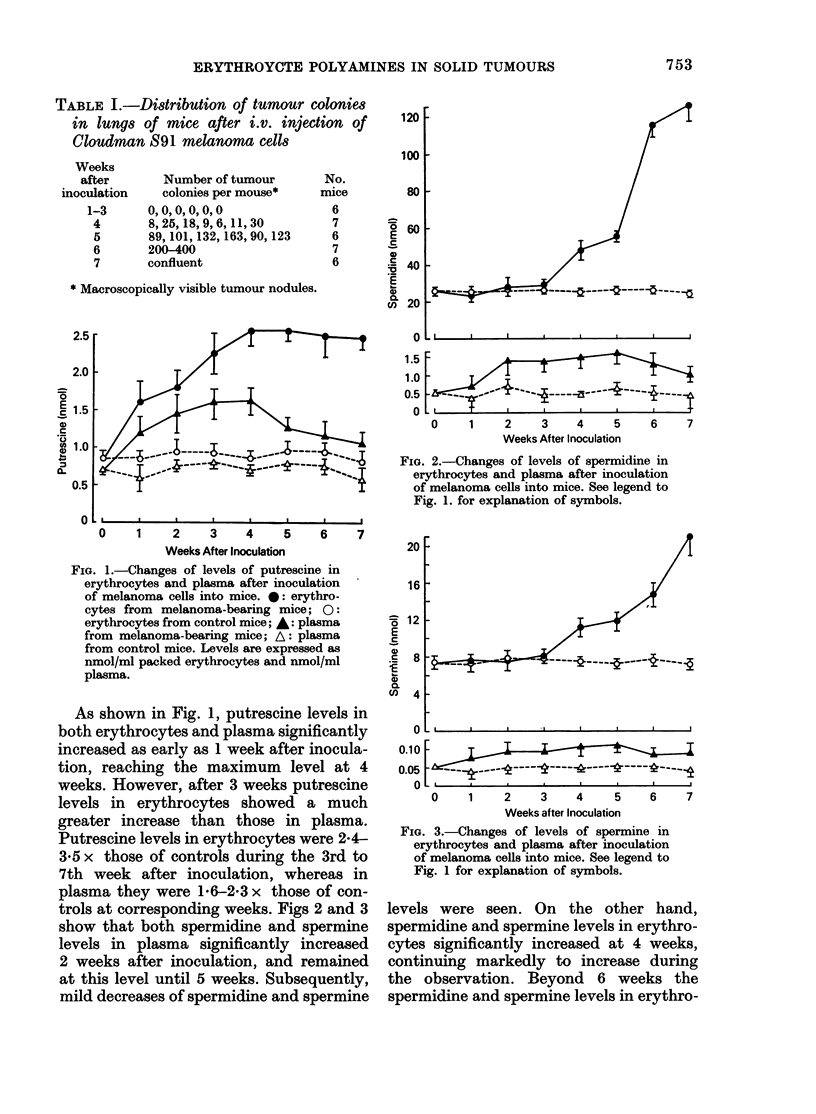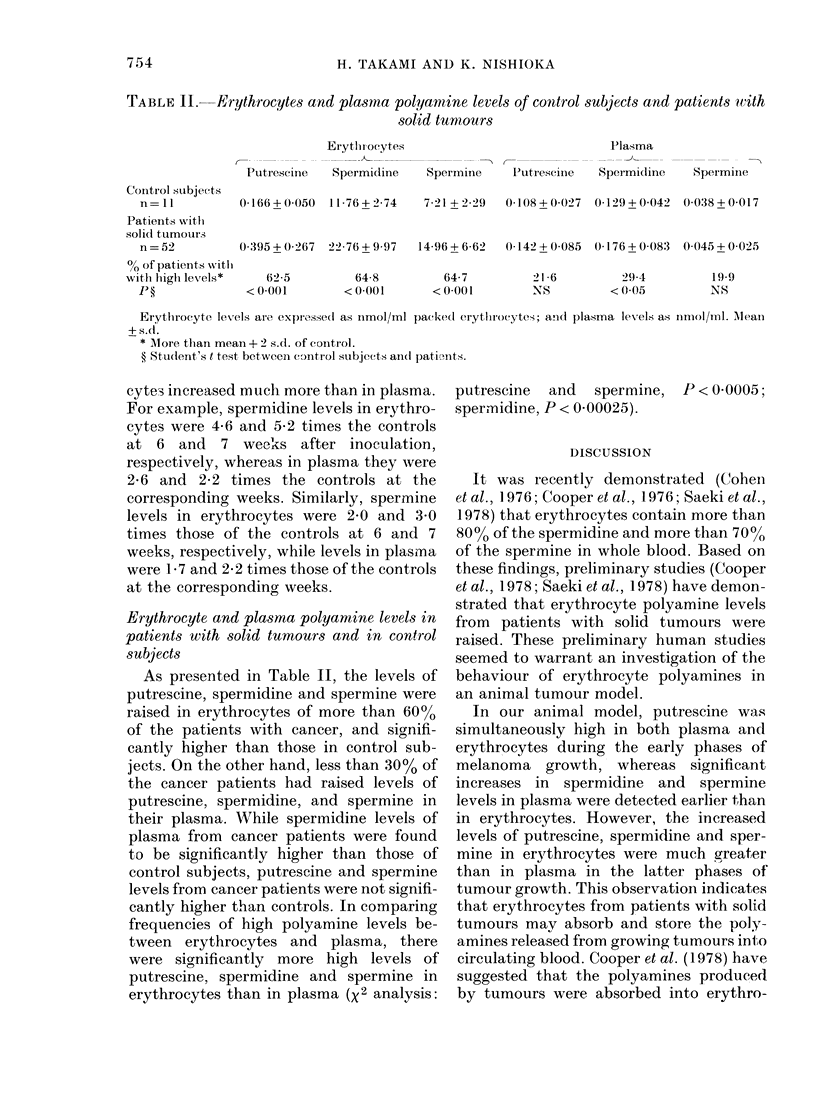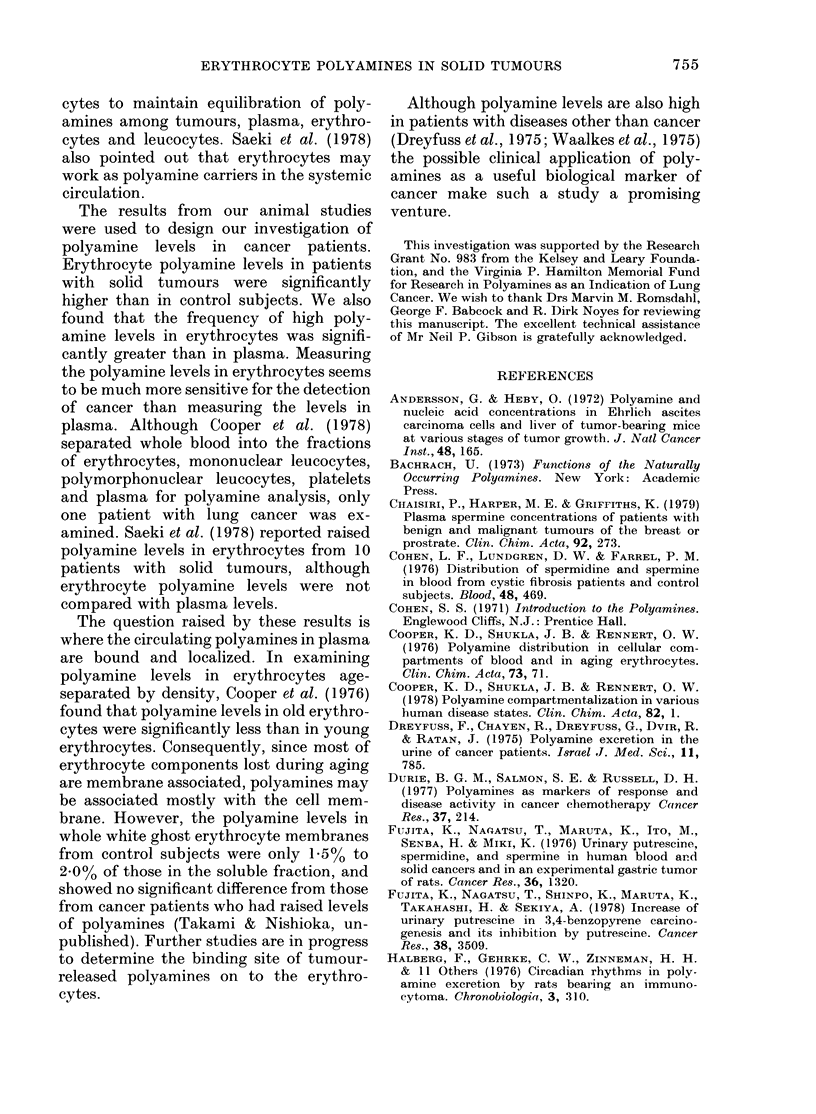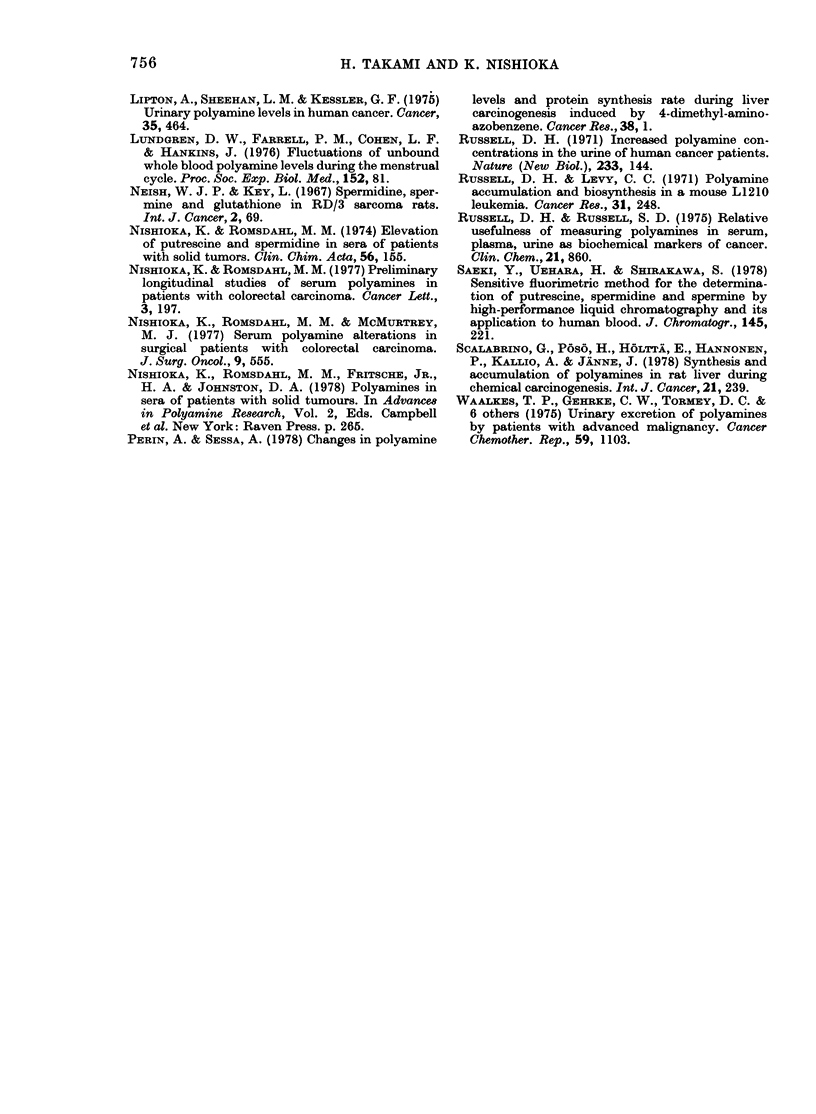# Raised polyamines in erythrocytes from melanoma-bearing mice and patients with solid tumours

**DOI:** 10.1038/bjc.1980.137

**Published:** 1980-05

**Authors:** H. Takami, K. Nishioka

## Abstract

The levels of polyamines (putrescine, spermidine and spermine) in erythrocytes and plasma were studied using Cloudman S-91 melanoma grown in the lungs of DBA/2 mice. Polyamine levels and the numbers of tumour-cell colonies in the lungs were determined at weekly intervals. Putrescine levels in both erythrocytes and plasma significantly increased 1 week after tumour inoculation. Three weeks after inoculation, however, putrescine levels in the erythrocytes showed a greater increase than those in plasma. Spermidine and spermine levels were initially high at 2 weeks in plasma and at 4 weeks in erythrocytes. However, by 6 weeks the spermidine levels showed a greater increase in erythrocytes than in plasma. These data suggest that erythrocytes may absorb and store polyamines released into the circulation.

This finding was subsequently applied to human studies. Fifty-two untreated patients with solid tumours were examined in the preoperative period. All erythrocyte polyamine levels from patients were significantly higher than those from control subjects. Plasma spermidine levels in patients were significantly higher than those in controls, whereas plasma putrescine and spermine levels showed no significant increase. The frequency of raised levels of putrescine, spermidine and spermine in erythrocytes was significantly greater than in plasma. These results suggest that polyamine levels in erythrocytes may provide useful information for the detection of cancer.


*Br. J. Cancer* (1980) **41**, 751

### RAISED POLYAMINES IN ERYTHROCYTES FROM MELANOMA-BEARING MICE AND PATIENTS WITH SOLID TUMOURS

H. TAKAMI AND K. NISHIOKA

*From the Department of Surgery/Surgical Research Laboratory, the University of Texas System Cancer Center, M. D. Anderson Hospital and Tumor Institute, Houston, Texas* 77030, *U.S.A.*

Received 15 November 1979 Accepted 21 January 1980

**Summary.—The levels of polyamines (putrescine, spermidine and spermine) in erythrocytes and plasma were studied using Cloudman S-91 melanoma grown in the lungs of DBA/2 mice. Polyamine levels and the numbers of tumour-cell colonies in the lungs were determined at weekly intervals. Putrescine levels in both erythrocytes and plasma significantly increased 1 week after tumour inoculation. Three weeks after inoculation, however, putrescine levels in the erythrocytes showed a greater increase than those in plasma. Spermidine and spermine levels were initially high at 2 weeks in plasma and at 4 weeks in erythrocytes. However, by 6 weeks the spermidine levels showed a greater increase in erythrocytes than in plasma. These data suggest that erythrocytes may absorb and store polyamines released into the circulation.**

**This finding was subsequently applied to human studies. Fifty-two untreated patients with solid tumours were examined in the preoperative period. All erythrocyte polyamine levels from patients were significantly higher than those from control subjects. Plasma spermidine levels in patients were significantly higher than those in controls, whereas plasma putrescine and spermine levels showed no significant increase. The frequency of raised levels of putrescine, spermidine and spermine in erythrocytes was significantly greater than in plasma. These results suggest that polyamine levels in erythrocytes may provide useful information for the detection of cancer.**

THE NATURALLY occurring polyamines are known to have important regulatory functions in growth and proliferation of cells (Cohen, 1971; Bachrach, 1973). Since Russell (1971) first reported that cancer patients excreted an increased amount of polyamines in their urine, a number of papers have been published on raised polyamine levels in urine (Dreyfuss *et al.*, 1975; Lipton *et al.*, 1975; Waalkes *et al.*, 1975) and plasma or serum (Nishioka & Romsdahl, 1974; Russell & Russell, 1975; Nishioka & Romsdahl, 1977; Nishioka *et al.*, 1977; Chaisiri *et al.*, 1979) of cancer patients. At present, however, the clinical application of polyamine determinations as a diagnostic tool for cancer detection has been limited by the low sensitivity and lack of specificity of polyamine detection in urine or plasma.

Recently, high polyamine levels have been found in erythrocytes from patients with cancer (Saeki *et al.*, 1978; Cooper *et* al., 1978). These reports dealt with a limited number of patients. At this time, very little information is available on the level of polyamines in erythrocytes during the cancer state in humans. Although some reports also indicated high levels of polyamines in tumour tissues or urine of tumour-transplanted animals (Neish & Key, 1967; Russell & Levy, 1971; Andersson & Heby, 1972) and during carcinogenesis (Fujita *et al.*, 1976, 1978; Perin & Sessa, 1978; Scalabrino *et al.*, 1978), no values have been reported for polyamines in whole blood of tumour-bearing animals. Therefore, using an animal model system, we investigated the fluctuation of polyamine levels in erythrocytes and plasma at various times after inoculation of melanoma cells. Based on these results, an attempt was made to study erythrocyte polyamine levels relative to plasma polyamine levels in cancer patients, and to examine the potential usefulness of erythrocyte polyamines as biological markers for cancer.

### MATERIALS AND METHODS

*Experimental mouse melanoma.*—Male inbred DBA/2 mice, aged 5 weeks (18–21 g) were purchased from Timco Breeding Laboratories (Houston, Texas, U.S.A.), and fed a standard laboratory diet *ad libitum*.

The melanoma cells used were maintained in tissue culture in Eagle's minimum essential medium (MEM) supplemented with 10% foetal calf serum (Gibco, Grand Island, N.Y., U.S.A.). One-tenth ml of the cultured cells ($10^6$ cells/ml) suspended in MEM was injected into the tail vein. Each week thereafter at least 6 mice were sacrificed to collect blood. The lungs were examined macroscopically for pigmented melanoma colonies, which were then counted. Control mice were injected with 0·1 ml of MEM and sacrificed in groups of 4 (minimum). Blood was drawn from retro-orbital plexus under ether anaesthesia and placed in heparinized tubes. All blood was collected between 10.00 and 11.00 to avoid difference due to circadian rhythm (Halberg *et al.*, 1976).

*Patients with solid tumours.*—The 52 (18 M; 34 F) preoperative patients examined (mean age, 59) with solid tumours (breast, 16; colorectum, 14; lung, 8; melanoma, 7; miscellaneous, 7) had no previous treatments, blood transfusions or other diseases. The control subjects were 11 (5 M; 6 F) healthy closely age-matched individuals (mean age, 55). Premenopausal females were excluded from this study due to known fluctuation in polyamine levels during the menstrual cycle (Lundgren *et al.*, 1976). Heparinized blood was collected during the fasting state in the morning.

*Preparation of samples and determination of polyamines.*—Whole blood was immediately centrifuged at 500 *g* for 20 min after collection. The plasma was then removed for polyamine assay, and the buffy coat leucocytes were carefully aspirated from the erythrocytes (Cooper, *et al.*, 1978; Saeki, *et al.*, 1978). Aliquots of plasma (0·3–0·35 ml from the mouse and 5·0 ml from the human) were used for polyamine analysis. The erythrocytes were subsequently washed with 3 volumes of cold physiological saline, centrifuged, and the buffy coat leucocytes aspirated as above. This procedure was repeated twice more. After thoroughly mixing the packed erythrocytes, aliquots of the erythrocyte fraction (0·2 ml from mouse sample and 1·0 ml from human sample) were used for preparation of polyamine samples.

Plasma and erythrocytes thus obtained were treated with trichloroacetic acid (TCA) as follows, according to the procedures of Durie *et al.* (1977) with some modifications. Plasma was mixed with 100% TCA to a final concentration of 5% TCA. An additional volume of 5% TCA (1·0 ml for mouse sample, 3·0 ml for human sample) was then added to the plasma. In the erythrocyte samples, 5% TCA solution was added to the erythrocytes first, followed by the 100% TCA, to avoid insufficient mixing which occurs if 100% TCA is added directly to erythrocytes. The samples were then mixed thoroughly for a minimum of 3 min and left standing at 4°C for 30 min. After centrifugation at 5,000 *g* for 20 min, the supernatants were collected and pellets re-extracted twice with 5% TCA as above. The supernatants were combined and lyophilized. These samples were hydrolysed in 6N HCl for 16 h at 104°C, extracted twice with ether, and again lyophilized. The residues were dissolved in 0·5 N HCl and analysed for polyamines, using a Durrum D-500 high-pressure amino acid analyser (Russell & Russell, 1975; Cooper *et al.*, 1976). The recoveries of putrescine, spermidine and spermine were 88·2%, 77·7% and 84·1%, respectively, in mice, whilst in humans recoveries were 85·0%, 78·4% and 86·4%, respectively.

### RESULTS

### *Erythrocyte and plasma polyamine levels in melanoma-bearing and normal mice*

In melanoma-bearing mice, no tumour colonies in the lungs were seen in the first 3 weeks after inoculation (Table I). Lung colonies appeared at 4 weeks, becoming confluent at 7 weeks. Metastases were not found, macroscopically or microscopically, in any other organs. All mice survived for at least 7 weeks after inoculation.

TABLE I.—*Distribution of tumour colonies in lungs of mice after i.v. injection of Cloudman S91 melanoma cells*

| Weeks after inoculation | Number of tumour colonies per mouse* | No. mice |
|---|---|---|
| 1–3 | 0, 0, 0, 0, 0, 0 | 6 |
| 4 | 8, 25, 18, 9, 6, 11, 30 | 7 |
| 5 | 89, 101, 132, 163, 90, 123 | 6 |
| 6 | 200–400 | 7 |
| 7 | confluent | 6 |

* Macroscopically visible tumour nodules.

FIG. 1.—Changes of levels of putrescine in erythrocytes and plasma after inoculation of melanoma cells into mice. ●: erythrocytes from melanoma-bearing mice; ○: erythrocytes from control mice; ▲: plasma from melanoma-bearing mice; △: plasma from control mice. Levels are expressed as nmol/ml packed erythrocytes and nmol/ml plasma.

FIG. 2.—Changes of levels of spermidine in erythrocytes and plasma after inoculation of melanoma cells into mice. See legend to Fig. 1. for explanation of symbols.

FIG. 3.—Changes of levels of spermine in erythrocytes and plasma after inoculation of melanoma cells into mice. See legend to Fig. 1 for explanation of symbols.

As shown in Fig. 1, putrescine levels in both erythrocytes and plasma significantly increased as early as 1 week after inoculation, reaching the maximum level at 4 weeks. However, after 3 weeks putrescine levels in erythrocytes showed a much greater increase than those in plasma. Putrescine levels in erythrocytes were 2·4–3·5 × those of controls during the 3rd to 7th week after inoculation, whereas in plasma they were 1·6–2·3 × those of controls at corresponding weeks. Figs 2 and 3 show that both spermidine and spermine levels in plasma significantly increased 2 weeks after inoculation, and remained at this level until 5 weeks. Subsequently, mild decreases of spermidine and spermine levels were seen. On the other hand, spermidine and spermine levels in erythrocytes significantly increased at 4 weeks, continuing markedly to increase during the observation. Beyond 6 weeks the spermidine and spermine levels in erythro-

TABLE II.—*Erythrocytes and plasma polyamine levels of control subjects and patients with solid tumours*

| | Erythrocytes | | | Plasma | | |
|---|---|---|---|---|---|---|
| | Putrescine | Spermidine | Spermine | Putrescine | Spermidine | Spermine |
| Control subjects n = 11 | 0·166 ± 0·050 | 11·76 ± 2·74 | 7·21 ± 2·29 | 0·108 ± 0·027 | 0·129 ± 0·042 | 0·038 ± 0·017 |
| Patients with solid tumours n = 52 | 0·395 ± 0·267 | 22·76 ± 9·97 | 14·96 ± 6·62 | 0·142 ± 0·085 | 0·176 ± 0·083 | 0·045 ± 0·025 |
| % of patients with with high levels* | 62·5 | 64·8 | 64·7 | 21·6 | 29·4 | 19·9 |
| *P*§ | < 0·001 | < 0·001 | < 0·001 | NS | < 0·05 | NS |

Erythrocyte levels are expressed as nmol/ml packed erythrocytes; and plasma levels as nmol/ml. Mean ± s.d.

* More than mean + 2 s.d. of control.

§ Student's *t* test between control subjects and patients.

cytes increased much more than in plasma. For example, spermidine levels in erythrocytes were 4·6 and 5·2 times the controls at 6 and 7 weeks after inoculation, respectively, whereas in plasma they were 2·6 and 2·2 times the controls at the corresponding weeks. Similarly, spermine levels in erythrocytes were 2·0 and 3·0 times those of the controls at 6 and 7 weeks, respectively, while levels in plasma were 1·7 and 2·2 times those of the controls at the corresponding weeks.

*Erythrocyte and plasma polyamine levels in patients with solid tumours and in control subjects*

As presented in Table II, the levels of putrescine, spermidine and spermine were raised in erythrocytes of more than 60% of the patients with cancer, and significantly higher than those in control subjects. On the other hand, less than 30% of the cancer patients had raised levels of putrescine, spermidine, and spermine in their plasma. While spermidine levels of plasma from cancer patients were found to be significantly higher than those of control subjects, putrescine and spermine levels from cancer patients were not significantly higher than controls. In comparing frequencies of high polyamine levels between erythrocytes and plasma, there were significantly more high levels of putrescine, spermidine and spermine in erythrocytes than in plasma ($\chi^2$ analysis: putrescine and spermine, $P < 0{\cdot}0005$; spermidine, $P < 0{\cdot}00025$).

### DISCUSSION

It was recently demonstrated (Cohen *et al.*, 1976; Cooper *et al.*, 1976; Saeki *et al.*, 1978) that erythrocytes contain more than 80% of the spermidine and more than 70% of the spermine in whole blood. Based on these findings, preliminary studies (Cooper *et al.*, 1978; Saeki *et al.*, 1978) have demonstrated that erythrocyte polyamine levels from patients with solid tumours were raised. These preliminary human studies seemed to warrant an investigation of the behaviour of erythrocyte polyamines in an animal tumour model.

In our animal model, putrescine was simultaneously high in both plasma and erythrocytes during the early phases of melanoma growth, whereas significant increases in spermidine and spermine levels in plasma were detected earlier than in erythrocytes. However, the increased levels of putrescine, spermidine and spermine in erythrocytes were much greater than in plasma in the latter phases of tumour growth. This observation indicates that erythrocytes from patients with solid tumours may absorb and store the polyamines released from growing tumours into circulating blood. Cooper *et al.* (1978) have suggested that the polyamines produced by tumours were absorbed into erythrocytes to maintain equilibration of polyamines among tumours, plasma, erythrocytes and leucocytes. Saeki *et al.* (1978) also pointed out that erythrocytes may work as polyamine carriers in the systemic circulation.

The results from our animal studies were used to design our investigation of polyamine levels in cancer patients. Erythrocyte polyamine levels in patients with solid tumours were significantly higher than in control subjects. We also found that the frequency of high polyamine levels in erythrocytes was significantly greater than in plasma. Measuring the polyamine levels in erythrocytes seems to be much more sensitive for the detection of cancer than measuring the levels in plasma. Although Cooper *et al.* (1978) separated whole blood into the fractions of erythrocytes, mononuclear leucocytes, polymorphonuclear leucocytes, platelets and plasma for polyamine analysis, only one patient with lung cancer was examined. Saeki *et al.* (1978) reported raised polyamine levels in erythrocytes from 10 patients with solid tumours, although erythrocyte polyamine levels were not compared with plasma levels.

The question raised by these results is where the circulating polyamines in plasma are bound and localized. In examining polyamine levels in erythrocytes age-separated by density, Cooper *et al.* (1976) found that polyamine levels in old erythrocytes were significantly less than in young erythrocytes. Consequently, since most of erythrocyte components lost during aging are membrane associated, polyamines may be associated mostly with the cell membrane. However, the polyamine levels in whole white ghost erythrocyte membranes from control subjects were only 1·5% to 2·0% of those in the soluble fraction, and showed no significant difference from those from cancer patients who had raised levels of polyamines (Takami & Nishioka, unpublished). Further studies are in progress to determine the binding site of tumour-released polyamines on to the erythrocytes.

Although polyamine levels are also high in patients with diseases other than cancer (Dreyfuss *et al.*, 1975; Waalkes *et al.*, 1975) the possible clinical application of polyamines as a useful biological marker of cancer make such a study a promising venture.

This investigation was supported by the Research Grant No. 983 from the Kelsey and Leary Foundation, and the Virginia P. Hamilton Memorial Fund for Research in Polyamines as an Indication of Lung Cancer. We wish to thank Drs Marvin M. Romsdahl, George F. Babcock and R. Dirk Noyes for reviewing this manuscript. The excellent technical assistance of Mr Neil P. Gibson is gratefully acknowledged.

### REFERENCES

Andersson, G. & Heby, O. (1972) Polyamine and nucleic acid concentrations in Ehrlich ascites carcinoma cells and liver of tumor-bearing mice at various stages of tumor growth. *J. Natl Cancer Inst.*, **48**, 165.

Bachrach, U. (1973) *Functions of the Naturally Occurring Polyamines.* New York: Academic Press.

Chaisiri, P., Harper, M. E. & Griffiths, K. (1979) Plasma spermine concentrations of patients with benign and malignant tumours of the breast or prostrate. *Clin. Chim. Acta*, **92**, 273.

Cohen, L. F., Lundgren, D. W. & Farrel, P. M. (1976) Distribution of spermidine and spermine in blood from cystic fibrosis patients and control subjects. *Blood*, **48**, 469.

Cohen, S. S. (1971) *Introduction to the Polyamines.* Englewood Cliffs, N.J.: Prentice Hall.

Cooper, K. D., Shukla, J. B. & Rennert, O. W. (1976) Polyamine distribution in cellular compartments of blood and in aging erythrocytes. *Clin. Chim. Acta*, **73**, 71.

Cooper, K. D., Shukla, J. B. & Rennert, O. W. (1978) Polyamine compartmentalization in various human disease states. *Clin. Chim. Acta*, **82**, 1.

Dreyfuss, F., Chayen, R., Dreyfuss, G., Dvir, R. & Ratan, J. (1975) Polyamine excretion in the urine of cancer patients. *Israel J. Med. Sci.*, **11**, 785.

Durie, B. G. M., Salmon, S. E. & Russell, D. H. (1977) Polyamines as markers of response and disease activity in cancer chemotherapy *Cancer Res.*, **37**, 214.

Fujita, K., Nagatsu, T., Maruta, K., Ito, M., Senba, H. & Miki, K. (1976) Urinary putrescine, spermidine, and spermine in human blood and solid cancers and in an experimental gastric tumor of rats. *Cancer Res.*, **36**, 1320.

Fujita, K., Nagatsu, T., Shinpo, K., Maruta, K., Takahashi, H. & Sekiya, A. (1978) Increase of urinary putrescine in 3,4-benzopyrene carcinogenesis and its inhibition by putrescine. *Cancer Res.*, **38**, 3509.

Halberg, F., Gehrke, C. W., Zinneman, H. H. & 11 Others (1976) Circadian rhythms in polyamine excretion by rats bearing an immunocytoma. *Chronobiologia*, **3**, 310.

LIPTON, A., SHEEHAN, L. M. & KESSLER, G. F. (1975) Urinary polyamine levels in human cancer. *Cancer*, **35**, 464.

LUNDGREN, D. W., FARRELL, P. M., COHEN, L. F. & HANKINS, J. (1976) Fluctuations of unbound whole blood polyamine levels during the menstrual cycle. *Proc. Soc. Exp. Biol. Med.*, **152**, 81.

NEISH, W. J. P. & KEY, L. (1967) Spermidine, spermine and glutathione in RD/3 sarcoma rats. *Int. J. Cancer*, **2**, 69.

NISHIOKA, K. & ROMSDAHL, M. M. (1974) Elevation of putrescine and spermidine in sera of patients with solid tumors. *Clin. Chim. Acta*, **56**, 155.

NISHIOKA, K. & ROMSDAHL, M. M. (1977) Preliminary longitudinal studies of serum polyamines in patients with colorectal carcinoma. *Cancer Lett.*, **3**, 197.

NISHIOKA, K., ROMSDAHL, M. M. & MCMURTREY, M. J. (1977) Serum polyamine alterations in surgical patients with colorectal carcinoma. *J. Surg. Oncol.*, **9**, 555.

NISHIOKA, K., ROMSDAHL, M. M., FRITSCHE, JR., H. A. & JOHNSTON, D. A. (1978) Polyamines in sera of patients with solid tumours. In *Advances in Polyamine Research*, Vol. 2, Eds. Campbell *et al.* New York: Raven Press. p. 265.

PERIN, A. & SESSA, A. (1978) Changes in polyamine levels and protein synthesis rate during liver carcinogenesis induced by 4-dimethyl-amino-azobenzene. *Cancer Res.*, **38**, 1.

RUSSELL, D. H. (1971) Increased polyamine concentrations in the urine of human cancer patients. *Nature* (*New Biol.*), **233**, 144.

RUSSELL, D. H. & LEVY, C. C. (1971) Polyamine accumulation and biosynthesis in a mouse L1210 leukemia. *Cancer Res.*, **31**, 248.

RUSSELL, D. H. & RUSSELL, S. D. (1975) Relative usefulness of measuring polyamines in serum, plasma, urine as biochemical markers of cancer. *Clin. Chem.*, **21**, 860.

SAEKI, Y., UEHARA, H. & SHIRAKAWA, S. (1978) Sensitive fluorimetric method for the determination of putrescine, spermidine and spermine by high-performance liquid chromatography and its application to human blood. *J. Chromatogr.*, **145**, 221.

SCALABRINO, G., PÖSÖ, H., HÖLTTÄ, E., HANNONEN, P., KALLIO, A. & JÄNNE, J. (1978) Synthesis and accumulation of polyamines in rat liver during chemical carcinogenesis. *Int. J. Cancer*, **21**, 239.

WAALKES, T. P., GEHRKE, C. W., TORMEY, D. C. & 6 others (1975) Urinary excretion of polyamines by patients with advanced malignancy. *Cancer Chemother. Rep.*, **59**, 1103.